# Giant bowing of the band gap and spin-orbit splitting energy in GaP_1−*x*_Bi_*x*_ dilute bismide alloys

**DOI:** 10.1038/s41598-019-43142-5

**Published:** 2019-05-02

**Authors:** Zoe L. Bushell, Christopher A. Broderick, Lukas Nattermann, Rita Joseph, Joseph L. Keddie, Judy M. Rorison, Kerstin Volz, Stephen J. Sweeney

**Affiliations:** 1 Advanced Technology Institute and Department of Physics, University of Surrey, Guildford, GU2 7XH UK; 2 Tyndall National Institute, Lee Maltings, Dyke Parade, Cork, T12 R5CP Ireland; 3 Department of Electrical and Electronic Engineering, University of Bristol, Bristol, BS8 1UB UK; 4 Materials Science Center and Faculty of Physics, Philipps-Universität Marburg, 35032 Marburg, Germany

**Keywords:** Electronic properties and materials, Semiconductors

## Abstract

Using spectroscopic ellipsometry measurements on GaP_1−*x*_Bi_*x*_/GaP epitaxial layers up to *x* = 3.7% we observe a giant bowing of the direct band gap (${E}_{g}^{{\rm{\Gamma }}}$) and valence band spin-orbit splitting energy (Δ_SO_). ${E}_{g}^{{\rm{\Gamma }}}$ (Δ_SO_) is measured to decrease (increase) by approximately 200 meV (240 meV) with the incorporation of 1% Bi, corresponding to a greater than fourfold increase in Δ_SO_ in going from GaP to GaP_0.99_Bi_0.01_. The evolution of ${E}_{g}^{{\rm{\Gamma }}}$ and Δ_SO_ with *x* is characterised by strong, composition-dependent bowing. We demonstrate that a simple valence band-anticrossing model, parametrised directly from atomistic supercell calculations, quantitatively describes the measured evolution of ${E}_{g}^{{\rm{\Gamma }}}$ and Δ_SO_ with *x*. In contrast to the well-studied GaAs_1−*x*_Bi_*x*_ alloy_,_ in GaP_1−*x*_Bi_*x*_ substitutional Bi creates localised impurity states lying energetically within the GaP host matrix band gap. This leads to the emergence of an optically active band of Bi-hybridised states, accounting for the overall large bowing of ${E}_{g}^{{\rm{\Gamma }}}$ and Δ_SO_ and in particular for the giant bowing observed for *x* ≲ 1%. Our analysis provides insight into the action of Bi as an isovalent impurity, and constitutes the first detailed experimental and theoretical analysis of the GaP_1−*x*_Bi_*x*_ alloy band structure.

## Introduction

Highly-mismatched III-V semiconductor alloys containing dilute concentrations of bismuth (Bi) have attracted significant attention in recent years^1^ since their unique electronic properties open up a range of possibilities for practical applications in semiconductor lasers^2–16^, photovoltaics^17,18^, spintronics^19–21^, photodiodes^22–25^, and thermoelectrics^26^. Research on dilute bismide alloys has primarily focused to date on GaAs_1−*x*_Bi_*x*_, where incorporation of Bi brings about a strong reduction of the direct Γ-point band gap (${E}_{g}^{{\rm{\Gamma }}}$)–by up to 90 meV per % Bi at low Bi compositions *x*^27–31^–characterised by strong, composition-dependent bowing^29,32^. This unusual behaviour derives from the large differences in size (covalent radius) and chemical properties (electronegativity) between As and Bi: Bi, being significantly larger and more electropositive than As, acts as an isovalent impurity which primarily impacts and strongly perturbs the valence band (VB) structure^30,33,34^. This is in contrast to dilute nitride alloys, in which small electronegative nitrogen (N) atoms strongly perturb the conduction band (CB) structure in GaN_*x*_As_1−*x*_ and related alloys^35–38^. Additionally Bi, being the largest stable group-V element, has strong relativistic (spin-orbit coupling) effects^39^. As such, the reduction of ${E}_{g}^{{\rm{\Gamma }}}$ in (In)GaAs_1−*x*_Bi_*x*_ is accompanied by a strong increase in the VB spin-orbit splitting energy (Δ_SO_)^16,30,31,40^.

Epitaxial growth of GaP_1−*x*_Bi_*x*_ alloys, via molecular beam epitaxy^41,42^ and metal-organic vapour phase epitaxy^43^ (MOVPE), has only recently been attempted. Here, we present the first detailed analysis of the GaP_1−*x*_Bi_*x*_ electronic band structure. Early experiments on impurities in GaP can be traced back to the advent of semiconductors, with the initial experiments of Trumbore *et al*.^44^ revealing that Bi dopants generate bound localised impurity states in GaP, i.e. Bi-related localised impurity states lying energetically within the GaP host matrix band gap. However, there is little further data available regarding the GaP_1−*x*_Bi_*x*_ band structure. Here, we explicitly verify that the evolution of the main features of the GaP_1−*x*_Bi_*x*_ VB structure with Bi composition *x* can be understood straightforwardly in terms of an *x*-dependent valence band-anticrossing (VBAC) interaction between the extended states of the GaP VB edge and localised bound impurity states associated with substitutional Bi impurities. The VBAC interaction between these extended and localised states produces a set of Bi-hybridised bands, with the GaP_1−*x*_Bi_*x*_ alloy VB edge then being a primarily Bi-derived band possessing an admixture of GaP VB edge Bloch character^30^, enabling optical coupling to the comparatively unperturbed Γ_6*c*_ CB states. Comparison between theory and experiment highlights the emergence of this Bi-derived impurity band lying energetically within the GaP band gap, close in energy to the unperturbed GaP VB edge. Our analysis reveals the giant bowing of ${E}_{g}^{{\rm{\Gamma }}}$ and Δ_SO_ due to this VBAC interaction: ${E}_{g}^{{\rm{\Gamma }}}$ (Δ_SO_) decreases (increases) by ≈200 meV (240 meV) when 1% Bi is incorporated substitutionally in GaP.

We begin here by describing our measurement procedure and the general features of our experimental results. Next, we describe our theoretical model, which is then applied to analyse the trends revealed by the experimental measurements. Finally, we use the results of this combined theoretical and experimental analysis to describe general features of the GaP_1−*x*_Bi_*x*_ band structure.

## Experimental Measurements

Spectroscopic ellipsometry (SE) was used to study bulk-like GaP_1−*x*_Bi_*x*_ epitaxial layers containing up to 3.7% Bi. The samples were grown on (001)-oriented GaP by MOVPE, with x-ray diffraction measurements confirming that the layers are in a state of compressive pseudomorphic strain. Full details of the sample growth and characterisation–including data from high-resolution x-ray diffraction, atomic force microscopy, secondary ion mass spectrometry, and scanning transmission electronic microscopy measurements–can be found in ref.^43^. The SE measurements were performed at room temperature using a J. A. Woollam Co. variable angle spectroscopic ellipsometer. Three incident beam angles were used to generate sufficient data to provide confidence in modelling fits to the measured spectra. Angles of 73.5°, 74.0° and 74.5° were chosen since they are close to the pseudo-Brewster angles of the samples under investigation, thereby ensuring that the phase change on reflection Δ (measured relative to the sample normal) remained close to 90°. When Δ is close to 0 or 180°, the level of noise in the measured SE spectra is increased, and the sensitivity of the ellipsometic parameters to small changes in the optical properties is decreased. Keeping Δ close to 90° increases both the precision and accuracy of the measurements^45^. A carefully defined modelling and fitting procedure was used to extract the energies corresponding to critical points in the band structure from the measured SE data, allowing the energies corresponding to the ${E}_{g}^{{\rm{\Gamma }}}$ and ${E}_{g}^{{\rm{\Gamma }}}+{{\rm{\Delta }}}_{{\rm{SO}}}$ inter-band transitions to be extracted^46^. Full details of this fitting procedure are presented as Supplementary Material.

**Figure 1:**
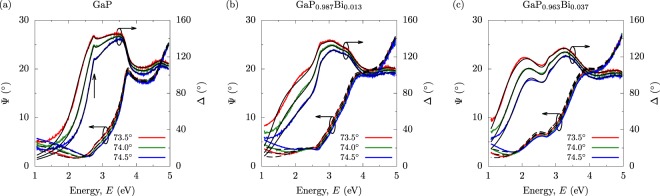
Measured SE spectra for the MOVPE-grown (**a**) GaP (Bi-free, *x* = 0), (**b**) GaP_0.987_Bi_0.013_ (*x* = 1.3%), and (**c**) GaP_0.963_Bi_0.037_ (*x* = 3.7%) samples described in the text and in ref.^43^. Solid red, green and blue lines respectively denote data measured for incident beam angles of 73.5°, 74.0° and 74.5°. Solid (dashed) black lines show the fits to the measured Δ (Ψ) spectra; the SE model and associated fitting procedure are outlined in the text.

The solid red, green and blue lines in Fig. 1(a–c) respectively show the measured SE data–where *tan*(Ψ) is related to the change in the ratio of the amplitudes of the *p*- and *s*-polarisations upon reflection^45^–in the GaP, GaP_0.987_Bi_0.013_ and GaP_0.963_Bi_0.037_ samples. Solid (dashed) black lines show the corresponding fits to Δ (Ψ). The GaP sample consists of an epitaxial GaP buffer layer grown on a GaP substrate, and was analysed first in order to obtain accurate input parameters for the SE fits. These parameters were then used to describe the substrate and buffer layer in the subsequent models of the Bi-containing samples. Following this procedure it was possible to achieve good fits to the key features observed in the measured GaP_1−*x*_Bi_*x*_ SE spectra (cf. Fig. 1(b,c)). In Fig. 1(a) a clear feature associated with ${E}_{g}^{{\rm{\Gamma }}}$ is visible in the measured Δ and Ψ spectra, which is well described by a modelling fit corresponding to a Γ-point GaP room temperature band gap ${E}_{g}^{{\rm{\Gamma }}}=2.76$ eV. The slight deviation from the accepted value of 2.78 eV is attributable to the sample growth taking place on n-doped GaP substrates^47^.

**Figure 2:**
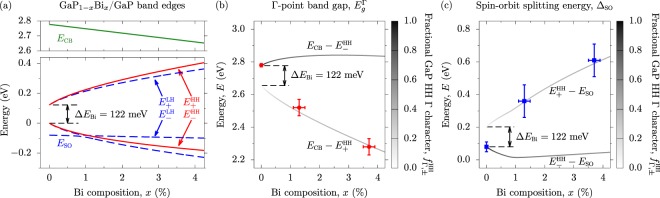
(**a**) Calculated variation of the Γ-point band edge energies with *x* in pseudomorphically strained GaP_1−*x*_Bi_*x*_/GaP. Solid green and red lines respectively denote the CB- and HH-like band edge energies, while dashed blue lines denote the LH- and SO-like band edge energies. (**b**) Variation of the GaP_1−*x*_Bi_*x*_/GaP Γ-point band gaps ${E}_{{\rm{CB}}}-{E}_{\pm }^{{\rm{HH}}}$ with *x*, calculated (shaded lines) and extracted from SE measurements (closed red circles). The line shading is determined by the fractional GaP HH Γ character ${f}_{{\rm{\Gamma }},\pm }^{{\rm{HH}}}$ of the associated HH-like GaP_1−*x*_Bi_*x*_ VB states ${E}_{\pm }^{{\rm{HH}}}$. (**c**) Variation of the GaP_1−*x*_Bi_*x*_/GaP VB spin-orbit splitting energies ${E}_{\pm }^{{\rm{HH}}}-{E}_{{\rm{SO}}}$ with *x*, calculated (shaded lines) and extracted from SE measurements (closed blue circles). The line shading is as in (**b**).

Turning to Fig. 1(b,c) we note from the measured Δ spectra that Bi incorporation gives rise to an additional feature at lower energy than ${E}_{g}^{{\rm{\Gamma }}}$ in GaP, which shifts to even lower energies with increasing *x*. This indicates a large reduction of ${E}_{g}^{{\rm{\Gamma }}}$, in agreement with theoretical predictions^30,48,49^. The spectral features associated with ${E}_{g}^{{\rm{\Gamma }}}$ are significantly broader in the Bi-containing samples than in GaP. This is likely associated with the presence of Bi composition fluctuations across the samples, as well as short-range alloy disorder, associated with the formation of pairs and larger clusters of Bi atoms sharing common Ga nearest neighbours in a substitutional alloy^50,51^. Using the fitting procedure outlined in the Supplementary Material it was also possible to extract the energies associated with the ${E}_{g}^{{\rm{\Gamma }}}+{{\rm{\Delta }}}_{{\rm{SO}}}$ transitions in each sample. The values of ${E}_{g}^{{\rm{\Gamma }}}$ and Δ_SO_ extracted in this manner are shown respectively in Fig. 2(b,c), using closed red circles and blue squares. We note that the uncertainties in these data are associated with the broadening of the corresponding features in the measured spectra^46^. Overall, the SE measurements indicate that incorporation of dilute concentrations of Bi is sufficient to cause a giant reduction (increase) and bowing of ${E}_{g}^{{\rm{\Gamma }}}$ (Δ_SO_).

## Theoretical Calculations

To understand this unusual behaviour we have used alloy supercell electronic structure calculations to analyse the contributions to the Bi-induced changes in the band edge energies, and to parametrise a suitable VBAC model for GaP_1−*x*_Bi_*x*_. This approach does not rely on post hoc fitting to alloy band structure data, thereby providing a predictive capability commonly lacking in models of this type^32^. In ref.^30^ we employed an atomistic tight-binding (TB) model to analyse the electronic structure of ordered and disordered GaP_1−*x*_Bi_*x*_ alloys. By directly constructing the *T*_2_-symmetric localised states |*ψ*_Bi_〉 associated with an isolated, substitutional Bi impurity we predicted the presence of a VBAC interaction having a composition dependence $\beta \sqrt{x}$. In the dilute doping (large supercell) limit we determined that the Bi-related localised states in GaP:Bi lie approximately 120 meV above the unperturbed GaP VB edge, in good agreement with experiment^44^. Analysis of the electronic structure of ordered Ga(P,As)_1−*x*_Bi_*x*_ alloys eludicates the differences in the impact of Bi incorporation on the band structure: the natural VB offsets between GaP, GaAs and GaBi lead to the *6p* valence orbitals of Bi lying below the *4p* valence orbitals of As in energy, but higher in energy than the *3p* valence orbitals of P. As such, a substitutional Bi impurity forms a resonant localised state lying energetically below the VB edge in GaAs, but a bound localised state lying above the VB edge in energy in GaP^30^.

Building on our initial analysis of GaP_1−*x*_Bi_*x*_ we have derived an extended basis set 12-band (VBAC) **k** ⋅ **p** Hamiltonian to describe the dilute bismide band structure^32^. Using the TB model of ref.^30^ we have directly evaluated the Bi-related parameters of this model, including the distinct VBAC, virtual crystal (VC) and strain-related contributions to the Bi-induced shifts in the band edge energies^52^. To analyse the SE measurements we focus on the band edge energies at the zone centre: at Γ the 12-band Hamiltonian diagonalises into decoupled blocks describing the CB, heavy-hole (HH), light-hole (LH) and spin-split-off (SO) band edges^52^. As in GaAs_1−*x*_Bi_*x*_, the energy of the GaP_1−*x*_Bi_*x*_ Γ-point CB state Γ_6*c*_ is well described as ${E}_{{\rm{CB}}}(x)={E}_{g}^{{\rm{\Gamma }}}\mathrm{(0)}-\alpha \,x+\delta {E}_{{\rm{CB}}}^{{\rm{hy}}}$, where the zero of energy has been chosen at the unperturbed GaP VB edge, ${E}_{g}^{{\rm{\Gamma }}}\mathrm{(0)}=2.78$ eV is the host matrix band gap, *α* describes the VC shift of the CB edge energy, and $\delta {E}_{{\rm{CB}}}^{{\rm{hy}}}$ is the energy shift associated with the hydrostatic component of the compressive pseudomorphic strain in a GaP_1−*x*_Bi_*x*_/GaP epitaxial layer^52^.

The energies of the HH-like alloy VB states are given in the 12-band VBAC model as the eigenvalues of the 2 × 2 matrix^30,52^

1 $$(\begin{array}{cc}{\rm{\Delta }}{E}_{{\rm{B}}{\rm{i}}}+\delta {E}_{{\rm{B}}{\rm{i}}}^{{\rm{h}}{\rm{y}}}-\delta {E}_{{\rm{B}}{\rm{i}}}^{{\rm{a}}{\rm{x}}} & \beta \sqrt{x}\\ \beta \sqrt{x} & \kappa \,x+\delta {E}_{{\rm{V}}{\rm{B}}}^{{\rm{h}}{\rm{y}}}-\delta {E}_{{\rm{V}}{\rm{B}}}^{{\rm{a}}{\rm{x}}}\end{array})\,\,\begin{array}{c}|{\psi }_{{\rm{B}}{\rm{i}}}^{{\rm{H}}{\rm{H}}}\rangle \\ |{\psi }_{{\rm{H}}{\rm{H}}}^{(0)}\rangle \end{array},$$

where $\kappa \,x+\delta {E}_{{\rm{VB}}}^{{\rm{hy}}}-\delta {E}_{{\rm{VB}}}^{{\rm{ax}}}$ describes the VC, hydrostatic and axial strain-induced shifts to the GaP HH band edge energy, and ${\rm{\Delta }}{E}_{{\rm{Bi}}}+\delta {E}_{{\rm{Bi}}}^{{\rm{hy}}}-\delta {E}_{{\rm{Bi}}}^{{\rm{ax}}}$ is the energy of the HH-like Bi-related localised states relative to the zero of energy at the unperturbed GaP VB edge^52^. The energies of the LH- and SO-like VB states are given as the eigenvalues of a 3 × 3 matrix which can be found, along with full details of the model, in ref.^52^.

The Bi-related band structure parameters computed for GaP_1−*x*_Bi_*x*_ are summarised in Table 1 where, for comparative purposes, the corresponding parameters computed for GaAs_1−*x*_Bi_*x*_ are provided. Comparing these two sets of VBAC parameters we firstly note that while a substitutional Bi impurity in GaAs leads to the formation of a resonant localised state lying 183 meV below the VB edge (Δ*E*_Bi_ < 0), in GaP it leads to the formation of a bound localised state lying 122 meV above the VB edge (Δ*E*_Bi_ > 0). In both cases the alloy VB edge consists of an admixture of extended (Bloch) and localised (Bi-related) character. However, in GaAs_1−*x*_Bi_*x*_ the alloy VB edge states are primarily GaAs-derived, retaining significant Bloch character, while in GaP_1−*x*_Bi_*x*_ the alloy VB edge states are primarily Bi-derived, having low overall Bloch character^30^. As we will describe in further detail below, this key difference describes the fundamentally distinct nature of the perturbed VB structure in GaAs_1−*x*_Bi_*x*_ and GaP_1−*x*_Bi_*x*_ alloys, and has significant consequences for the electronic and optical properties. We note that this behaviour is qualitatively similar to the stark differences in the CB structure of the dilute nitride alloys GaN_*x*_As_1−*x*_ and GaN_*x*_P_1−*x*_: a substitutional N impurity in GaAs creates a N-related localised state which is resonant with the CB, while in GaP it creates a bound impurity state lying energetically within the band gap^35,38,53,54^.

**Table 1:** Bi-related parameters for the 12-band (VBAC) **k** ⋅ **p** Hamiltonian of Ga(P,As)_1−*x*_Bi_*x*_, computed using atomistic TB calculations on ordered alloy supercells. The energy Δ*E*_Bi_ of the Bi-related localised impurity states is given relative to the unperturbed Ga(P,As) host matrix VB edge^30,32,52^.

Parameter	GaP_1−*x*_Bi_*x*_	GaAs_1−*x*_Bi_*x*_
Δ*E*_Bi_ (eV)	0.122	−0.183
*α* (eV)	4.39	2.82
*β* (eV)	1.41	1.13
*γ* (eV)	0.24	0.55
*κ* (eV)	1.47	1.01

Considering the VBAC coupling parameter *β*, we note that a larger value is calculated for GaP_1−*x*_Bi_*x*_. This describes that the VBAC interaction is more pronounced in GaP_1−*x*_Bi_*x*_ than in GaAs_1−*x*_Bi_*x*_–i.e. that a substitutional Bi impurity more strongly perturbs the VB structure in GaP than in GaAs–reflecting the larger differences in size and electronegativity between P and Bi than between As and Bi. We note that *β* in GaP_1−*x*_Bi_*x*_ is comparable to that calculated previously for the GaN_*x*_P_1−*x*_ CB (*β* = 1.74 eV)^38,54^. Turning our attention to the VC contributions to the band offsets–described via the parameters *α*, *κ* and *γ* for the CB, VB and SO offsets respectively –we again note that the calculated differences in these parameters for GaP_1−*x*_Bi_*x*_ and GaAs_1−*x*_Bi_*x*_ alloys reflect the associated trends in the lattice mismatch and natural band offsets between GaP, GaAs and the fictitious semimetallic zinc blende compound GaBi^30,32,33,55^. The calculated values *α* = 4.39 and 2.82 eV for GaP_1−*x*_Bi_*x*_ and GaAs_1−*x*_Bi_*x*_ respectively describe increases of approximately 44 and 28 meV per % Bi of the type-I zone-centre CB offset in free-standing GaP_1−*x*_Bi_*x*_/GaP and GaAs_1−*x*_Bi_*x*_/GaAs, reflecting the larger difference in energy between the Γ_6*c*_ CB edge states in GaP and GaBi compared to that between GaAs and GaBi. For the VB offset, the larger calculated value of *κ* for GaP_1−*x*_Bi_*x*_ describes the larger VB offset between GaP and GaBi than between GaAs and GaBi. Similarly, the smaller calculated value of *γ* for GaP_1−*x*_Bi_*x*_ describes the smaller SO band offset between GaP and GaBi than between GaAs and GaBi, and results from a combination of (i) a natural VB offset between GaP and GaAs which exceeds the difference in Δ_SO_ between the two compounds^56^, and (ii) the extremely large predicted VB spin-orbit splitting in GaBi^39^.

Finally, since the III-V compound AlAs has approximately the same natural VB offset relative to GaAs as GaP^56^, we note that the VB structure of the AlAs_1−*x*_Bi_*x*_ alloy–growth of which has also recently been established^57^–is expected to be qualitatively the same as in GaP_1−*x*_Bi_*x*_. That is, based on the known chemical trends for III-V compounds, it can be expected based on the analysis presented here that (i) a substitutional Bi impurity in AlAs leads to the formation of a bound Bi-related localised impurity state, having the same general character as that described here in GaP, and (ii) this leads in turn to a large decrease (increase) and composition-dependent bowing of ${E}_{g}^{{\rm{\Gamma }}}$ (Δ_SO_) at dilute Bi compositions *x*^58^.

## Results

Figure 2(a) shows the calculated variation of the Γ-point band edge energies with *x* in pseudomorphically strained GaP_1−*x*_Bi_*x*_/GaP, for the CB (*E*_CB_, solid black line), HH (${E}_{\pm }^{{\rm{HH}}}$, solid red lines), LH (${E}_{\pm }^{{\rm{LH}}}$, dashed blue lines) and SO (*E*_SO_, dash-dotted green lines) states, respectively. The hydrostatic component of the pseudomorphic strain pushes the CB (HH, LH and SO) edge(s) upwards (downwards) in energy, while the axial component lifts the degeneracy of VB edge in the usual manner^59,60^. Since the GaP_1−*x*_Bi_*x*_/GaP epitaxial layers under investigation are in a state of compressive pseudomorphic strain, this splitting of the HH- and LH-like states leads in general to HH-like VB edge eigenstates^52^. We calculate that *E*_CB_ reduces linearly with increasing *x*, by 18 meV per % Bi. The VBAC interaction produces two Bi-hybridised HH-like bands, the energies ${E}_{\pm }^{{\rm{HH}}}$ of which vary strongly with *x*, displaying strong composition-dependent bowing. Beginning from ${E}_{-}^{{\rm{HH}}}=0$ and ${E}_{+}^{{\rm{HH}}}={\rm{\Delta }}{E}_{{\rm{Bi}}}$ at *x* = 0, we calculate that ${E}_{-}^{{\rm{HH}}}$ (${E}_{\pm }^{{\rm{HH}}}$) decreases (increases) by 79 meV (103 meV) between *x* = 0 and 1%. Similarly, the VBAC interaction produces a set of LH- and SO-like Bi-hybridised bands^52^, the energies of which are again strongly dependent on *x* and characterised by strong composition-dependent bowing. As ${E}_{\pm }^{{\rm{LH}}}$ moves downwards in energy towards *E*_SO_ with increasing *x* the coupling between the LH- and SO-like states–which is brought about by the axial component of the pseudomorphic strain^52^–leads to an anticrossing which is manifested in an abrupt increase in the rate at which *E*_SO_ decreases for $x\gtrsim 1$%. We note also that this axial strain-induced anticrossing between the LH- and SO-like states leads to a change of the VB ordering at Γ, with ${E}_{-}^{{\rm{LH}}} > {E}_{-}^{{\rm{HH}}}$ for $x\gtrsim 1$%.

Since the SE measurements do not detect optically forbidden transitions, such as those between the Γ_6*c*_ CB edge states and the Bi-related localised states^32,61^, quantitative assessment of the evolution of ${E}_{g}^{{\rm{\Gamma }}}$ and Δ_SO_ with Bi composition *x* depends critically on the character of the hybridised GaP_1−*x*_Bi_*x*_/GaP alloy VB edge eigenstates. As described above, since the GaP_1−*x*_Bi_*x*_/GaP epitaxial layers under investigation are in a state of compressive pseudomorphic strain, the highest energy alloy VB states are expected to be HH-like (cf. Fig. 2(a)). It is therefore sufficient to investigate the character of the HH-like alloy eigenstates $|{\psi }_{\pm }^{{\rm{HH}}}\rangle ={a}_{{\rm{HH}}}^{(\pm )}\,|{\psi }_{{\rm{HH}}}^{\mathrm{(0)}}\rangle +{a}_{{\rm{Bi}}}^{(\pm )}\,|{\psi }_{{\rm{Bi}}}^{{\rm{HH}}}\rangle$ of Eq. (1)–corresponding respectively to the eigenvalues ${E}_{\pm }^{{\rm{HH}}}$–which are formed of a linear combination of the extended HH band edge state $|{\psi }_{{\rm{HH}}}^{\mathrm{(0)}}\rangle$ of the unperturbed GaP host matrix, and the HH-like Bi-related localised state $|{\psi }_{{\rm{Bi}}}^{{\rm{HH}}}\rangle$.

Since Δ*E*_Bi_ > 0 in GaP_1−*x*_Bi_*x*_, the higher energy ${E}_{+}^{{\rm{HH}}}$ eigenstate $|{\psi }_{+}^{{\rm{HH}}}\rangle$ of Eq. (1) is primarily Bi-derived $(|{a}_{{\rm{HH}}}^{(+)}{|}^{2} < \frac{1}{2})$. Furthermore, given that the Bi-related localised states $|{\psi }_{{\rm{Bi}}}^{{\rm{HH}}}\rangle$ do not couple optically to the Γ_6*c*_ CB edge states^32^, any optical transitions between $|{\psi }_{+}^{{\rm{HH}}}\rangle$ and the Γ-point CB edge, having energy ${E}_{{\rm{CB}}}-{E}_{+}^{{\rm{HH}}}$, result from the VBAC interaction imparting GaP HH fractional Γ character ${f}_{{\rm{\Gamma }},+}^{{\rm{HH}}}\equiv |\langle {\psi }_{{\rm{HH}}}^{\mathrm{(0)}}|{\psi }_{+}^{{\rm{HH}}}\rangle {|}^{2}=|{a}_{{\rm{HH}}}^{(+)}{|}^{2}$ to $|{\psi }_{+}^{{\rm{HH}}}\rangle$. Using Eq. (1), ${f}_{{\rm{\Gamma }},+}^{{\rm{HH}}}$ can be determined analytically as

2 $${f}_{{\rm{\Gamma }},+}^{{\rm{HH}}}=\frac{{\beta }^{2}x}{{\beta }^{2}x+{({E}_{+}^{{\rm{HH}}}-\kappa x-\delta {E}_{{\rm{VB}}}^{{\rm{hy}}}+\delta {E}_{{\rm{VB}}}^{{\rm{ax}}})}^{2}}.$$

As *x* increases the increase in the strength $\beta \sqrt{x}$ of the VBAC interaction leads to $|{\psi }_{+}^{{\rm{HH}}}\rangle$ acquiring significant GaP HH Γ character which, despite being limited to values $< \frac{1}{2}$, is sufficient to produce appreciable optical coupling to the Γ_6*c*_ CB states. At *x* = 1% we calculate ${f}_{{\rm{\Gamma }},+}^{{\rm{HH}}}=0.315$, indicating that the optical transition strength between $|{\psi }_{+}^{{\rm{HH}}}\rangle$ ($|{\psi }_{-}^{{\rm{HH}}}\rangle$) and Γ_6*c*_ in an *ordered* GaP_0.99_Bi_0.01_ alloy should be close to one-third (two-thirds) of that between Γ_8*v*_ and Γ_6*c*_ in GaP. Thus, our analysis indicates the emergence of a hybridised alloy VB edge in the form of an impurity band: this band has (i) energy ${E}_{+}^{{\rm{HH}}}$ and has primarily Bi-related localised character, and (ii) optical coupling to the comparatively unperturbed Γ_6*c*_ CB edge states which increases with increasing Bi composition *x* (equivalently, decreasing Bi localised character, $1-{f}_{{\rm{\Gamma }},+}^{{\rm{HH}}}$).

To reflect this admixture of GaP (Bloch) and Bi (localised) character we have calculated the four distinct energy gaps ${E}_{{\rm{CB}}}-{E}_{\pm }^{{\rm{HH}}}$ and ${E}_{\pm }^{{\rm{HH}}}-{E}_{{\rm{SO}}}$. These energies represent the distinct separations evolving from the GaP direct band gap ${E}_{g}^{{\rm{\Gamma }}}$ and VB spin-orbit splitting energy Δ_SO_, as a direct result of the VBAC-induced hybridisation producing states having GaP HH character at distinct energies ${E}_{\pm }^{{\rm{HH}}}$. The results of these calculations are shown, using shaded lines, in Fig. 2(b,c)–for ${E}_{g}^{{\rm{\Gamma }}}$ and Δ_SO_, respectively–where they are compared to the values of ${E}_{g}^{{\rm{\Gamma }}}$ and Δ_SO_ extracted from the SE measurements of Fig. 1(a–c). To describe the optical activity of these transitions the lines denoting the calculated transition energies are shaded according to the GaP HH Γ character ${f}_{{\rm{\Gamma }},\pm }^{{\rm{H}}{\rm{H}}}$ of the corresponding HH-like alloy VB edge states $|{\psi }_{\pm }^{{\rm{HH}}}\rangle$, with solid black describing a purely GaP-like state having ${f}_{{\rm{\Gamma }},-}^{{\rm{HH}}}\equiv |{a}_{{\rm{HH}}}^{(-)}{|}^{2}=1$.

The SE measurements are sensitive to optically allowed inter-band transitions, and are hence capable in principle of detecting transitions between (i) the CB edge and the HH-like VBs, having transition energies ${E}_{{\rm{CB}}}-{E}_{\pm }^{{\rm{HH}}}$, and (ii) the CB edge and the SO band, having transition energy *E*_CB_ − *E*_SO_. No clear features were distinguishable in the measured SE spectra close to the calculated transition energy ${E}_{{\rm{CB}}}-{E}_{-}^{{\rm{HH}}}$. However, we note that the calculated ${E}_{{\rm{CB}}}-{E}_{-}^{{\rm{HH}}}$ transition energy for each sample is close to the *E*_CB_ − *E*_SO_ transition energy associated with the SO band. On the basis of the SE measurements it was not possible to determine whether there exist two distinct transitions in this energy range, although the presence of such transitions may explain the large spectral linewidths required to describe the measured SE spectra in this energy range (as reflected in the large estimated errors in the extracted values of Δ_SO_).

The quantitative agreement between the calculated and measured data in Fig. 2(b,c) confirms that the extremely large observed reduction (increase) and bowing of ${E}_{g}^{{\rm{\Gamma }}}$ (Δ_SO_) results from the emergence of an optically active band of primarily Bi-derived impurity states lying energetically within the GaP band gap. That this impurity band lies within the GaP band gap accounts quantitatively for the observed trends: the contribution of the strong, composition-dependent bowing of ${E}_{+}^{{\rm{HH}}}$ to the decrease (increase) of ${E}_{g}^{{\rm{\Gamma }}}$ (Δ_SO_) is combined with the binding energy Δ*E*_Bi_ of the Bi-related localised states. This behaviour is qualitatively distinct from that in GaAs_1−*x*_Bi_*x*_, where substitutional Bi atoms generate localised states which are resonant with the GaAs VB^30,33,62,63^, but similar to that in dilute nitride GaN_*x*_P_1−*x*_, where substitutional N atoms produce a band of primarily N-derived states lying deep within the GaP band gap^37,38,53,54,64–67^.

From the SE measurements we extract ${E}_{g}^{{\rm{\Gamma }}}=2.52$ eV at *x* = 1.3% (${f}_{{\rm{\Gamma }},+}^{{\rm{HH}}}=0.338$), an extremely large reduction of 240 meV compared to the measured GaP Γ-point band gap ${E}_{g}^{{\rm{\Gamma }}}\mathrm{(0)=2.78}$ eV. This is in good agreement with the calculated reduction of 284 meV in ${E}_{g}^{{\rm{\Gamma }}}$ between *x* = 0 and 1.3% in a pseudmorphically strained, ordered GaP_1−*x*_Bi_*x*_/GaP alloy. Given the calculated reduction of 38 meV in *E*_CB_ between *x* = 0 and 1.3%, we conclude that the majority (87%) of the reduction in ${E}_{g}^{{\rm{\Gamma }}}$ is associated with the emergence of the ${E}_{+}^{{\rm{HH}}}$ impurity band. Similarly, we measure an extremely large (>fourfold) increase of Δ_SO_, from 80 meV in GaP to approximately 360 meV at *x* = 1.3%. This is again in excellent agreement with the calculated value Δ_SO_ = 355 meV, with the majority (92%) of the increase in Δ_SO_ associated with the emergence of the ${E}_{+}^{{\rm{HH}}}$ band.

Increasing *x* from 1.3 to 3.7% we note that the change in ${E}_{g}^{{\rm{\Gamma }}}$ and Δ_SO_ per % Bi is significantly reduced. The measured (calculated) value ${E}_{g}^{{\rm{\Gamma }}}\mathrm{=2.28}$ eV (2.289 eV) at *x* = 3.7% (${f}_{{\rm{\Gamma }},+}^{{\rm{HH}}}=0.421$) represents a further reduction of 240 meV (207 meV) from that at *x* = 1.3%, while the measured (calculated) value Δ_SO_ = 1 eV (0.590 eV) at *x* = 3.7% Bi represents a further increase of 250 meV (235 meV) from that at *x* = 1.3%. For ${E}_{g}^{{\rm{\Gamma }}}$ this change is only 73% of that between *x* = 0 and 1.3%, despite occuring over a 2.4% increase in *x*. The measured and calculated changes of Δ_SO_ between *x* = 1.3 and 3.7% are approximately equal to those between *x* = 0 and 1.3%, again representing a significantly reduced change per % Bi. These trends highlight the strong dependence of the bowing of ${E}_{g}^{{\rm{\Gamma }}}$ and Δ_SO_ on *x*. Our measured and calculated variation of ${E}_{g}^{{\rm{\Gamma }}}$ and Δ_SO_ with *x* differs from that predicted using first principles electronic structure calculations^48^, but is close to that calculated via a VBAC model using parameter estimates based on available data for related alloys^49^.

We now turn our attention to two key qualitative features of the GaP_1−*x*_Bi_*x*_ electronic structure. Firstly, GaP has an indirect band gap due to the X_6*c*_ CB states lying ≈0.5 eV below Γ_6*c*_, while in semimetallic GaBi the X_6*c*_ states lie ≈2 eV above Γ_6*c*_^30,55^. Applying the VC approximation in conjunction with the TB model we estimate that the X_6*c*_ states shift downwards in energy by ≈12 meV per % Bi in free-standing GaP_1−*x*_Bi_*x*_. This is less than the 44 meV per % Bi reduction of the Γ_6*c*_ state energy described by the VC parameter *α* (cf. Table 1), suggesting that Bi incorporation may bring about a direct band gap for sufficiently high *x*. Based on the calculated evolution of the GaP_1−*x*_Bi_*x*_ CB structure with *x* (cf. Supplementary Material) we estimate that a direct band gap exists for high Bi compositions $x\gtrsim 30$%, suggesting that a direct band gap cannot be achieved at Bi compositions which are compatible with epitaxial growth. This highlights an important qualitative difference between the GaP_1−*x*_Bi_*x*_ and GaN_*x*_P_1−*x*_ band structures, since substitutional N in GaP generates localised states lying below the host matrix X_6*c*_ states in energy, bringing about a quasi-direct band gap even at ultra-dilute N compositions^37,53,54,64–66^.

Secondly, our analysis in ref.^30^ demonstrated that the VBAC description of the GaP_1−*x*_Bi_*x*_ VB structure breaks down with increasing *x* in the presence of short-range alloy disorder. Bi clustering creates a distribution of Bi-related localised states with which the GaP VB edge states strongly hybridise. This leads to a distribution of GaP VB edge Γ character over a multiplicity of impurity levels, suggesting that there is no single band possessing sufficient Bloch character to allow for appreciable absorption or emission of light^30^. While our results above demonstrate that the VBAC model provides a useful approach to analyse the main features of the band structure, the details of the electronic structure are in practice determined primarly by the impact of short-range alloy disorder. The difficulty in obtaining photoluminescence from the samples studied here, despite their high crystalline quality^43^, supports this interpretation: the GaP_1−*x*_Bi_*x*_ optical properties are intrinsically limited not solely by growth-related defects commonly associated with Bi incorporation, but by a combination of the indirect band gap and breakdown in VB edge Bloch character.

Despite having lattice constants commensurate with growth on Si, our analysis suggests that refinement of the epitaxial growth of GaP_1−*x*_Bi_*x*_ alloys is unlikely to lead to efficient light emitters: the optical properties are expected to be intrinsically limited by the nature of the material band structure. However, just as quaternary GaN_*x*_As_*y*_P_1−*x*−*y*_ alloys have found applications in III-V semiconductor lasers monolithically integrated on Si^68^, it is possible that similar progress could be made using As-rich quaternary GaP_1−*x*−*y*_As_*y*_Bi_*x*_ alloys for, e.g., applications in multi-junction solar cells, due to the fact that these alloys can be grown lattice-matched to either GaAs or germanium (Ge) while having band gaps close to 1 eV^69–71^.

## Conclusion

In conclusion, we have presented a combined experimental and theoretical investigation of the GaP_1−*x*_Bi_*x*_ band structure. Measurements performed on GaP_1−*x*_Bi_*x*_/GaP epitaxial layers reveal giant bowing of ${E}_{g}^{{\rm{\Gamma }}}$ and Δ_SO_, whereby ${E}_{g}^{{\rm{\Gamma }}}$ (Δ_SO_) decreases (increases) by approximately 200 meV (240 meV) between *x* = 0 and 1%. These changes are characterised by strong, composition-dependent bowing. Electronic structure calculations confirm that substitutional Bi in GaP generates localised impurity states lying energetically within the GaP band gap, and that the main features of the GaP_1−*x*_Bi_*x*_ band structure can be understood in terms of a VBAC interaction between the extended states of the GaP VB edge, and highly localised Bi-related impurity states. A VBAC model was derived and parametrised directly from atomistic supercell calculations, allowing quantitative prediction of the evolution of the main features of the band structure with *x*. Our analysis suggests that the highest energy VB in GaP_1−*x*_Bi_*x*_ is a hybridised impurity band: admixture of the GaP VB edge Γ character into this primarily Bi-derived band allows optical coupling to the comparatively unperturbed CB states. Aspects of the GaP_1−*x*_Bi_*x*_ band structure are broadly comparable to GaN_*x*_P_1−*x*_, but key qualitative differences highlight the distinction between Bi and N as isovalent impurities in conventional III-V semiconductors.

## Supplementary information

Supplementary material
