# Supplementary material for: Giant bowing of the band gap and spin-orbit splitting energy in GaP1−xBix dilute bismide alloys
Source: Sci Rep. 2019 May 2;9:6835. doi: 10.1038/s41598-019-43142-5 (PMC6497675; doi:10.1038/s41598-019-43142-5)
Supplement: Supplementary file 1 — Supplementary material [file 41598_2019_43142_MOESM1_ESM.pdf]

Supplementary material for "Giant bowing of the band gap and spin-orbit splitting energy in $GaP_{1-x}Bi_x$ dilute bismide alloys"

Zoe L. Bushell[1], Christopher A. Broderick[2,3,*], Lukas Nattermann[4], Rita Joseph[1], Joseph L. Keddie[1], Judy M. Rorison[3], Kerstin Volz[4], and Stephen J. Sweeney[1,†]

[1]Advanced Technology Institute and Department of Physics, University of Surrey, Guildford GU2 7XH, U.K.
[2]Tyndall National Institute, Lee Maltings, Dyke Parade, Cork T12 R5CP, Ireland
[3]Department of Electrical and Electronic Engineering, University of Bristol, Bristol BS8 1UB, U.K.
[4]Materials Science Center and Faculty of Physics, Philipps-Universität Marburg, 35032 Marburg, Germany
[*]c.broderick@umail.ucc.ie
[†]s.sweeney@surrey.ac.uk

Experimental measurements and data analysis

The spectroscopic ellipsometry (SE) measurements described in the main text were performed using a variable-angle spectroscopic ellipsometer (VASE; J. A. Woollam Co., Lincoln, Nebaska, USA). The VASE instrument employs a xenon lamp as a light source and a silicon detector, allowing for the generation and detection of light having wavelengths ranging from the ultraviolet to the near-infrared (approximately in the range 300 – 1100 nm). A double-beam monochromator is used to select the required wavelength for analysis, allowing full spectral measurements across this wavelength range. The monochromatic light generated in this manner is passed through a polariser with a known orientation and directed onto the sample surface. After reflection from the sample surface the beam (now elliptically polarised) passes through a rotating polariser – called the analyser – and onto the detector. The amount of light passing through the analyser, which determines the strength of the detector signal, varies as a function of the relative angle between the analyser and the polarisation of the reflected beam. Measuring the variation of the detector signal as a function of this relative angle can therefore be used to determine the polarisation of the reflected light. This information is then compared to the known polarisation of the incident beam in order to determine the change in polarisation caused by reflection at the sample surface, allowing the two SE parameters Ψ and Δ to be determined as a function of wavelength for a particular angle of incidence .[1–3]

As described in the main text, spectral data were collected for three different angles of incidence of the light beam (measured from the sample normal): 73.5°, 74.0° and 74.5°. These angles of incidence were selected on the basis that they are close to the pseudo-Brewster angles of the samples under investigation. These incident angles ensured that the value Δ of the phase change upon reflection from the sample surface is close to 90°, which corresponds to the range of maximum sensitivity for the VASE configuration.

The measured SE parameters Ψ and Δ were fit using a parametrised physical model based on that of Kim and Garland.[5] This model represents the complex dielectric function of a zincblende semiconductor as the sum of individual Gaussian oscillators, each of which is described by a transition energy, amplitude and spectral broadening. In our analysis of the measured SE data we employ a model which includes five Gaussian oscillators, corresponding to the following inter-band transitions: (i) between the Γ_{8v} VB and Γ_{6c} CB states, corresponding to the zone-centre direct band gap E_g^{Γ}, (ii) between the Γ_{7v} SO and Γ_{6c} CB states, corresponding to the sum of the zone-centre direct band gap and VB spin-orbit splitting energy $E_g^{\Gamma} + \Delta_{\text{SO}}$, (iii) between the highest energy (HH-like) VB and lowest energy CB along the Λ direction in the Brillouin zone, having energy E_1, (iv) between the SO VB and lowest energy CB along the Λ direction in the Brillouin zone, having energy $E_1 + \Delta_1$, and (v) between the Γ_{8v} VB and $\Gamma_{7,8c}$ CB states, having energy E_0'.[5] A description of the lineshapes associated with the spectral "tails" located energetically on either side of a given critical point (Gaussian oscillator) energy is included in the model, and was not varied when fitting to the measured Ψ and Δ data.[3,4] This parametrised model of the complex dielectric function enforces Kramers-Kronig consistency on the dielectric function, and has been validated for compound semiconductor films having both direct and indirect band gaps.[6–8] The model was taken to include a native oxide layer on the surface of each sample, which was described using tabulated optical data.[9] The thickness of each epitaxial layer was determined independently via high-resolution x-ray diffraction (HR-XRD) measurements,[10] and used as input to the model, i.e. the layer thickness was not used as a fitting parameter in the parametrised Gaussian oscillator model of the dielectric function for a given sample.

Table 1. Details of the parametrised fits to the measured SE data, for the $GaP_{1-x}Bi_x$ samples investigated in the main text. For each sample five Gaussian oscillators are used to fit to the measured SE data: the best-fit amplitude, critical point energy and spectral broadening associated with each oscillator is listed. The layer thicknesses used as input for the fits to the measured SE data, and the mean squared error (MSE) for the fit to each sample, are also provided.

Sample	Oscillator	Amplitude	Energy (eV)	Broadening (meV)	Thickness (nm)	MSE
GaP	E_g^Γ	0.17	2.76	20	Bulk	0.018
	$E_g^\Gamma + \Delta_{SO}$	0.11	2.84	46		
	E_1	17.0	3.70	65		
	$E_1 + \Delta_1$	9.9	3.74	51		
	E_0'	19.0	4.89	130		
$GaP_{0.987}Bi_{0.013}$	E_g^Γ	0.001	2.52	69	66	0.037
	$E_g^\Gamma + \Delta_{SO}$	0.90	2.85	105		
	E_1	17.0	3.70	65		
	$E_1 + \Delta_1$	9.9	3.74	51		
	E_0'	19.0	4.89	130		
$GaP_{0.963}Bi_{0.037}$	E_g^Γ	0.02	2.28	1740	50	0.036
	$E_g^\Gamma + \Delta_{SO}$	4.6	2.91	354		
	E_1	17.0	3.70	65		
	$E_1 + \Delta_1$	9.9	3.74	51		
	E_0'	19.0	4.89	130		

For each sample investigated an iterative fitting procedure was implemented using the J. A. Woollam VASE for Windows software, in order to minimise the difference between the measured and simulated SE data. The quality of the fit was measured via the mean squared error (MSE) between the measured and fitted Ψ and Δ spectra. The MSE was minimised at each iteration using a Marquardt-Levenberg fitting algorithm, in order to find the best-fit oscillator amplitudes, critical point energies, and spectral broadening.[11] The fitting procedure for each sample proceeded as follows. Firstly, since we were concerned only with dilute Bi compositions, the starting parameters for the Gaussian oscillator model for each $GaP_{1-x}Bi_x$ sample were chosen as those provided in the VASE software for GaP – i.e. using well-established literature values for GaP. Secondly, the amplitudes for all five Gaussian oscillators were fit to the measured SE data. Thirdly, the energies associated with each of the critical points were fit to the measured SE data. Finally, using the amplitudes and energies obtained in this manner, the spectral broadening associated with each critical point was determined by an overall best fit to the measured SE data.

The critical point energies obtained following this approach then provided the E_g^Γ and $E_g^\Gamma + \Delta_{SO}$ inter-band transition energies for each sample. The fitting procedure was benchmarked by application to GaAs, where the extracted critical point energies were found to be in excellent agreement with the well-established values from the literature.[3,4] For the Bi-containing samples, we note that the parameters resulting from this fitting procedure are comparable to those obtained in previous analysis using a critical point parabolic band model.[3] Table 1 summarises the best-fit parameters obtained for each of the $GaP_{1-x}Bi_x$ samples, including the amplitudes, critical point energies and spectral broadening, as well as the input layer thicknesses and resulting MSE associated with the overall fit to the Ψ and Δ spectra.

As an example of the optical constants of the $GaP_{1-x}Bi_x$ samples obtained using this approach, Figs. 1(a) and 1(b) respectively show the extracted real and imaginary parts n and κ of the complex refractive index $n + i\kappa$, for the same $GaP_{1-x}Bi_x$ samples investigated in the main text.[3] Examining the measured data for the imaginary part κ of the refractive index in Fig. 1(b) we note the significant reduction in energy of the absorption edge with increasing Bi composition x, consistent with the large reduction in the direct band gap E_g^Γ brought about by Bi incorporation.

This approach was applied to measure data for each of the samples described in Ref.[10], up to a maximum Bi composition $x = 8.3\%$. However, for $x > 3.7\%$ we found that it was not possible to identify sufficiently well defined features in the associated SE spectra to enable extraction of critical point energies, and hence the direct band gap E_g^Γ and spin-orbit splitting energy Δ_{SO}.[3] Atomic force and transmission electron microscopy performed on these higher Bi composition samples indicated phase separation due to the formation of Bi-rich regions – effectively metallic droplets of pure Bi – on the surface and in the interior of the $GaP_{1-x}Bi_x$ epitaxial layers, suggesting a breakdown of substitutional Bi incorporation.[10] It is possible that this phase separation, and the accompanying degradation in crystalline quality, is responsible for the inability to obtain a reliable set of SE data for the samples having $x \approx 7 – 8\%$.

The observed features associated with the E_g^Γ and $E_g^\Gamma + \Delta_{SO}$ inter-band transitions in the measured SE spectra for $GaP_{1-x}Bi_x$ alloys up to $x = 3.7\%$ have significantly larger spectral linewidths than those observed in SE measurements performaed on the

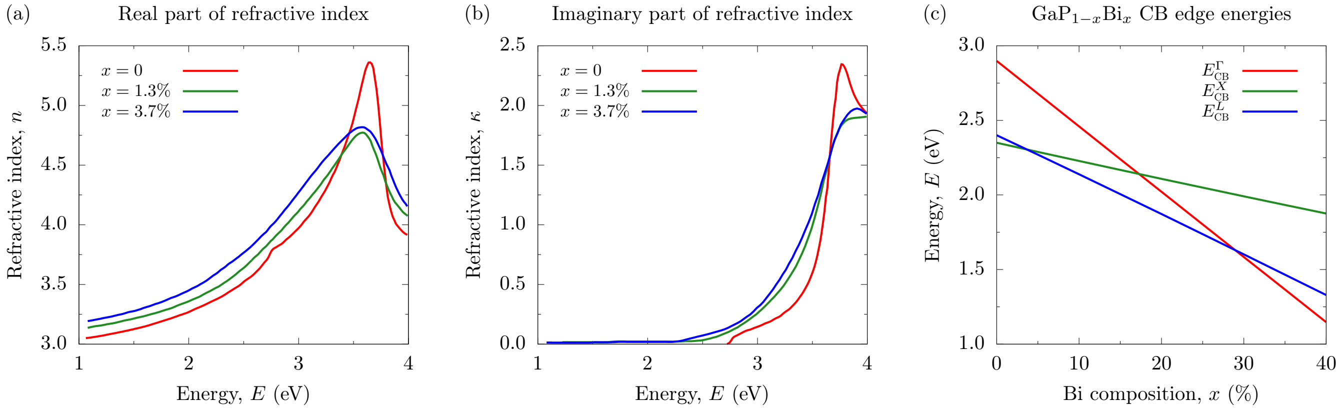

Figure 1. Measured (a) real, and (b) imaginary parts, n and κ, of the complex refractive index $n + i\kappa$ of the GaP (Bi-free; solid red lines), $GaP_{0.987}Bi_{0.013}$ ($x = 1.3\%$; solid green lines), and $GaP_{0.963}Bi_{0.037}$ ($x = 3.7\%$; solid blue lines) samples described in the text and in Ref.[10]. (c) Calculated variation with Bi composition x of the energies E_{CB}^{Γ}, E_{CB}^{X} and E_{CB}^{L} of the lowest CB at the Γ, X and L points in the Brillouin zone. The zero of energy is maintained fixed at the VB edge for each value of x. A direct-gap alloy is achieved for $x \gtrsim 27.8\%$.

As- and N-containing dilute bismide alloys $GaAs_{1-x}Bi_x$, $GaN_yAs_{1-x-y}Bi_x$ and $GaP_{1-x-y}As_yBi_x$.[4] As described in the main text, we note that this is consistent the our theoretical calculations for disordered $GaP_{1-x}Bi_x$ alloys, and is predicted to be a consequence of the strong breakdown in Bloch character associated with hybridisation of extended GaP valence band (VB) states with a multiplicity of Bi-related localised impurity states.[12]

Theoretical calculations: indirect- vs. direct-gap

While substitutionally incorporated Bi atoms act as isovalent impurities in $Ga(P,As)_{1-x}Bi_x$, their associated localised states primarily impact the VB structure while leaving the conduction band (CB) structure relatively unperturbed. Indeed, previous analysis has demonstrated that the CB states in Bi-containing alloys can be relatively well described using the virtual crystal (VC) approximation, as in a conventional (non-highly-mismatched) semiconductor alloy. As such, in order to probe the transition from an indirect to direct band gap in $GaP_{1-x}Bi_x$ alloys we have used the TB model of Ref.[12] to calculate the CB structure in the VC approximation. For a given Bi composition x this consists of linearly interpolating the orbital energies E_α ($\alpha = s_{c,a}$, $p_{c,a}$, $s^*_{c,a}$), inter-atomic interaction matrix elements V_δ ($\delta = ss\sigma$, $pp\sigma$, $pp\pi$, $s_c p_a \sigma$, $s_a p_c \sigma$, $s^*_c p_a \sigma$, $s^*_a p_c \sigma$), as well as the atomic spin-orbit coupling matrix elements $\Delta_{c,a}$, between those of GaP and GaBi.

We have used this approach to calculate the evolution with x of the energies of the lowest energy CB states in free-standing (unstrained) $GaP_{1-x}Bi_x$ at the Γ, X and L points in the Brillouin zone: Γ_{6c}, X_{6c} and L_{6c}. The results of these calculations are summarised in Fig. 1(c), which shows the calculated variation with x of the energies E_{CB}^{Γ}, E_{CB}^{X} and E_{CB}^{L} of the Γ_{6c}, X_{6c} and L_{6c} states. The zero of energy is maintained fixed at the VB edge at each value of x. As described in the main text, the energy of the Γ_{6c} state reduces linearly with increasing x as $E_{CB}^{\Gamma}(x) = E_{CB}^{\Gamma}(0) - \alpha x$, where $E_{CB}^{\Gamma}(0) = 2.780$ eV is the zone-centre CB edge energy in GaP and $\alpha = 4.39$ eV describes that E_{CB}^{Γ} reduces by approximately 44 meV per % Bi in free-standing $GaP_{1-x}Bi_x$. Turning our attention to the indirect X_{6c} and L_{6c} CB minima, we again calculate linear decreases in energy with increasing x. Specifically, for the X_{6c} states we calculate $E_{CB}^{X}(x) = E_{CB}^{X}(0) - \alpha_X x$, with $E_{CB}^{X}(0) = 2.151$ eV and $\alpha_X = 1.23$ eV (decreasing by approximately 12 meV per % Bi), while for the L_{6c} states we calculate $E_{CB}^{L}(x) = E_{CB}^{L}(0) - \alpha_L x$, with $E_{CB}^{L}(0) = 2.202$ eV and $\alpha_L = 2.60$ eV (decreasing by approximately 26 meV per % Bi).

As such, our calculations suggest that Bi incorporation reduces the energy of the lowest CB at the Γ, X and L points, with the reduction in energy per % Bi being significantly larger for Γ_{6c} than for either of X_{6c} or L_{6c}, and slightly larger for L_{6c} than for X_{6c}. On the basis of these trends we calculate the presence of (i) an indirect band gap for low Bi compositions $\lesssim 4\%$, with the lowest energy CB state being the X_{6c} state, (ii) an indirect band gap for Bi compositions $4\% \lesssim x \lesssim 28\%$, with the lowest energy CB state being the L_{6c} state, and (iii) a direct band gap for Bi compositions $\gtrsim 28\%$, with the lowest energy CB state being the Γ_{6c} state. We note here qualitative differences between these trends and those identified on the basis of the first principles calculations of Ref.[13], which suggested that the indirect Γ_{8v}-X_{6c} band gap reduces more strongly with increasing x than the direct Γ_{8v}-Γ_{6c} band gap and hence it is not possible to achieve a direct band gap at any composition in $GaP_{1-x}Bi_x$. However, given (i) that the calculations in Ref.[13] were performed only for dilute Bi compositions $x \lesssim 4\%$, and (ii) that the X_{6c} states lie higher in energy than the Γ_{6c} states in first principles calculations for GaBi,[14] it is to be expected – in line with our analysis here – that an indirect- to direct-gap transition should occur for sufficiently high x.

Given the difficulty associated with incorporating Bi compositions $x \gtrsim 8\%$ (where phase separation has been observed in initial MOVPE-grown samples for $x \gtrsim 7\%$),[10] as well as the metastability of $GaP_{1-x}Bi_x$ alloys, we note that the high Bi compositions required to bring about a direct band gap are expected to be significantly higher than those which are likely achievable via epitaxial growth. As such, we predict that $GaP_{1-x}Bi_x$ alloys retain an indirect band gap at Bi compositions compatable with epitaxial growth. The presence of strain in the material, pseudomorphic or otherwise, will introduce quantitative modifications to these conclusions and will – in general, assuming compressive strain related to growth either on GaP or Si substrates – act to increase the Bi composition at which a direct gap is achieved, putting direct gap $GaP_{1-x}Bi_x$ alloys further beyond the reach of epitaxial growth.

References

1. H. Fujiwara, *Spectroscopic Ellipsometry: Principles and Applications*, John Wiley & Sons, Chichester (2007)
2. J. A. Woollam Co., *A Short Course in Ellipsometry* (2001)
3. Z. L. Bushell, Ph.D. thesis, University of Surrey (2017)
4. Z. L. Bushell, R. M. Joseph, L. Nattermann, P. Ludewig, K. Volz, J. L. Keddie, and S. J. Sweeney, J. Appl. Phys. **123**, 045701 (2018)
5. C. C. Kim, J. W. Garland, H. Abad, and P. M. Raccah, Phys. Rev. B **45**, 11749 (1992)
6. B. Johs, C. M. Herzinger, J. H. Dinan, A. Cornfield, and J. D. Benson, Thin Solid Films **313-314**, 137 (1998)
7. C. M. Herzinger, B. Johs, W. A. McGahan, and J. A. Woollam, J. Appl. Phys. **83**, 3323 (1998)
8. Y. S. Ihn, T. J. Kim, T. H. Ghong, Y. D. Kim, D. E. Aspnes, and J. Kossut, Thin Solid Films **455-456**, 222 (2004)
9. S. Zollner, Appl. Phys. Lett. **63**, 2523 (1993)
10. L. Nattermann, A. Beyer, P. Ludewig, T. Hepp, E. Sterzer, and K. Volz, J. Cryst. Growth **463**, 151 (2017)
11. D. W. Marquardt, J. Soc. Ind. Appl. Math. **11**, 431 (1963)
12. M. Usman, C. A. Broderick, A. Lindsay, and E. P. O'Reilly, Phys. Rev. B **84**, 245202 (2011)
13. M. P. Polak, P. Scharoch, and R. Kudraweic, Semicond. Sci. Technol. **30**, 094001 (2015)
14. A. Janotti, S.-H. Wei, and S. B. Zhang, Phys. Rev. B **65**, 115203 (2002)
